# Genetic defects of *GDF6 *in the zebrafish *out of sight *mutant and in human eye developmental anomalies

**DOI:** 10.1186/1471-2156-11-102

**Published:** 2010-11-11

**Authors:** Anneke I den Hollander, Janisha Biyanwila, Peter Kovach, Tanya Bardakjian, Elias I Traboulsi, Nicola K Ragge, Adele Schneider, Jarema Malicki

**Affiliations:** 1 Division of Craniofacial and Molecular Genetics, Tufts University, 136 Harrison Ave., Boston MA 02111, USA; 2 Department of Ophthalmology, Radboud University Nijmegen Medical Centre, Nijmegen 6525 GA, the Netherlands; 3 Clinical Genetics, Albert Einstein Medical Center, Philadelphia, Pennsylvania 19141, USA; 4 Center for Genetic Eye Diseases, Cole Eye Institute, Cleveland Clinic Foundation, Cleveland, Ohio 44195, USA; 5 Department of Physiology, Anatomy and Genetics, University of Oxford, Oxford OX1 3QX, UK; 6 Moorfields Eye Hospital, London, EC1V 2PD, UK

## Abstract

**Background:**

The size of the vertebrate eye and the retina is likely to be controlled at several stages of embryogenesis by mechanisms that affect cell cycle length as well as cell survival. A mutation in the zebrafish *out of sight *(*out*) locus results in a particularly severe reduction of eye size. The goal of this study is to characterize the *out*^*m233 *^mutant, and to determine whether mutations in the *out *gene cause microphthalmia in humans.

**Results:**

In this study, we show that the severe reduction of eye size in the *out*^*m233 *^mutant is caused by a mutation in the zebrafish *gdf6a *gene. Despite the small eye size, the overall retinal architecture appears largely intact, and immunohistochemical studies confirm that all major cell types are present in *out*^*m233 *^retinae. Subtle cell fate and patterning changes are present predominantly in amacrine interneurons. Acridine orange and TUNEL staining reveal that the levels of apoptosis are abnormally high in *out*^*m233 *^mutant eyes during early neurogenesis. Mutation analysis of the *GDF6 *gene in 200 patients with microphthalmia revealed amino acid substitutions in four of them. In two patients additional skeletal defects were observed.

**Conclusions:**

This study confirms the essential role of GDF6 in the regulation of vertebrate eye size. The reduced eye size in the zebrafish *out*^*m233 *^mutant is likely to be caused by a transient wave of apoptosis at the onset of neurogenesis. Amino acid substitutions in *GDF6 *were detected in 4 (2%) of 200 patients with microphthalmia. In two patients different skeletal defects were also observed, suggesting pleitrophic effects of *GDF6 *variants. Parents carrying these variants are asymptomatic, suggesting that *GDF6 *sequence alterations are likely to contribute to the phenotype, but are not the sole cause of the disease. Variable expressivity and penetrance suggest a complex non-Mendelian inheritance pattern where other genetic factors may influence the outcome of the phenotype.

## Background

Microphthalmia, anophthalmia, and chorioretinal coloboma are ocular malformations that affect 1 in 3000-4000 individuals [1-3]. In microphthalmia and anophthalmia, one or both eyes are abnormally small or clinically absent. Colobomata are clefts caused by absent eye tissue, due to a failure of the optic fissure to close. Colobomata are frequently grouped with microphthalmia and anophthalmia, as they are often associated with a reduction of eye size. The aetiology of these ocular malformations is complex, and a wide variation is seen in phenotypic expression. Recessive, dominant and X-linked modes of inheritance have been described, but often sporadic and non-Mendelian inheritance patterns are seen [4,5]. In addition, a variety of environmental factors may be causative in certain cases, including viral infection, such as rubella, irradiation and drug intake in pregnancy.

Mutations in the transcription factor *SOX2 *are the most prevalent monogenic cause of microphthalmia and anophthalmia identified to date [6]. Other genes include the transcription factors *PAX6*, *OTX2*, *CHX10 *and *RAX *[7-10]. More recently, mutations in three members of the transforming growth factor-β (TGF-β) superfamily (*BMP4*; *GDF6*, also known as *BMP13*; and *GDF3*) have been associated with microphthalmia/anophthalmia [11-15]. Members of the TGF-β superfamily of secretory signaling molecules play essential roles in embryonic development [16,17]. Members of this superfamily regulate cell proliferation and apoptosis, and play important roles in various processes such as the creation of dorsal-ventral axes in the embryo, specification of the neural crest, bone formation, and organogenesis [18-20].

A segmental deletion encompassing the *GDF6 *gene, and several amino acid substitutions in GDF6 have been identified in patients with ocular anomalies, including coloboma and microphthalmia [13-15]. In addition, a chromosomal rearrangement and several amino acid substitutions in *GDF6 *have been detected in patients with Klippel-Feil syndrome, a complex skeletal disorder characterized by congenital fusion of the cervical spine, and in patients with other skeletal defects [21,14]. Gdf6 is expressed in the dorso-temporal retina, and morpholino (MO) knockdown experiments of *Gdf6 *in zebrafish and Xenopus result in reduced eye size or even the absence of optic lobes [13,22]. As these experiments were performed using antisense compounds, it is likely that this variability reflects imperfections of the morpholino knockdown approach. In Xenopus, and to a lesser extent in zebrafish, this phenotype is accompanied by a disorganization of retinal layering [22,13]. In addition, the presence of skeletal anomalies (curled or kinked tails) was observed in a fraction of MO-treated zebrafish embryos. The loss of *Gdf6 *in homozygous knockout mice causes abnormalities in joint, ligament and cartilage formation, and variable ocular phenotypes [23,14]. Finally, a small eye phenotype in the zebrafish mutant *dark half*^*s327 *^was attributed to a nonsense mutation in the *gdf6a *gene [24]. The authors of this work show that *gdf6a *establishes dorsal-ventral positional information in the retina and controls the formation of the retinotectal map.

Given conflicting results of studies in different vertebrate model systems, the role of GDF6 in eye development merits further investigation. We performed analysis of the *gdf6a *gene in the zebrafish *out*^*m233 *^mutant, characterized by a severe reduction of eye size [25,26]. We found that the *out*^*m233 *^mutation eliminates the initiation codon in the *gdf6a *gene, disrupting the function of this gene. Despite the reduction of eye size, retinal lamination is normal in most mutant animals, and the optic nerve appears largely unaffected. The small eye size phenotype in *out*^*m233 *^is associated with a wave of apoptosis during early stages of development. Other than eye size reduction, no obvious defects are observed in the external appearance of *out*^*m233 *^mutants, including its craniofacial skeleton. *out*^*m233 *^mutant homozygotes frequently survive to adulthood and display a variable reduction of size. Other than eye defects, we have not observed obvious abnormalities in the appearance of swimming behavior of *out*^*m233 *^mutant homozygotes. To investigate the role of GDF6 in human eye defects, we screened the *GDF6 *gene in 200 patients suffering from microphthalmia, anophthalmia or related abmormalities, and detected amino acid substitutions in four of them.

## Results

### The *out of sight*^*m233 *^phenotype

**Figure 1:**
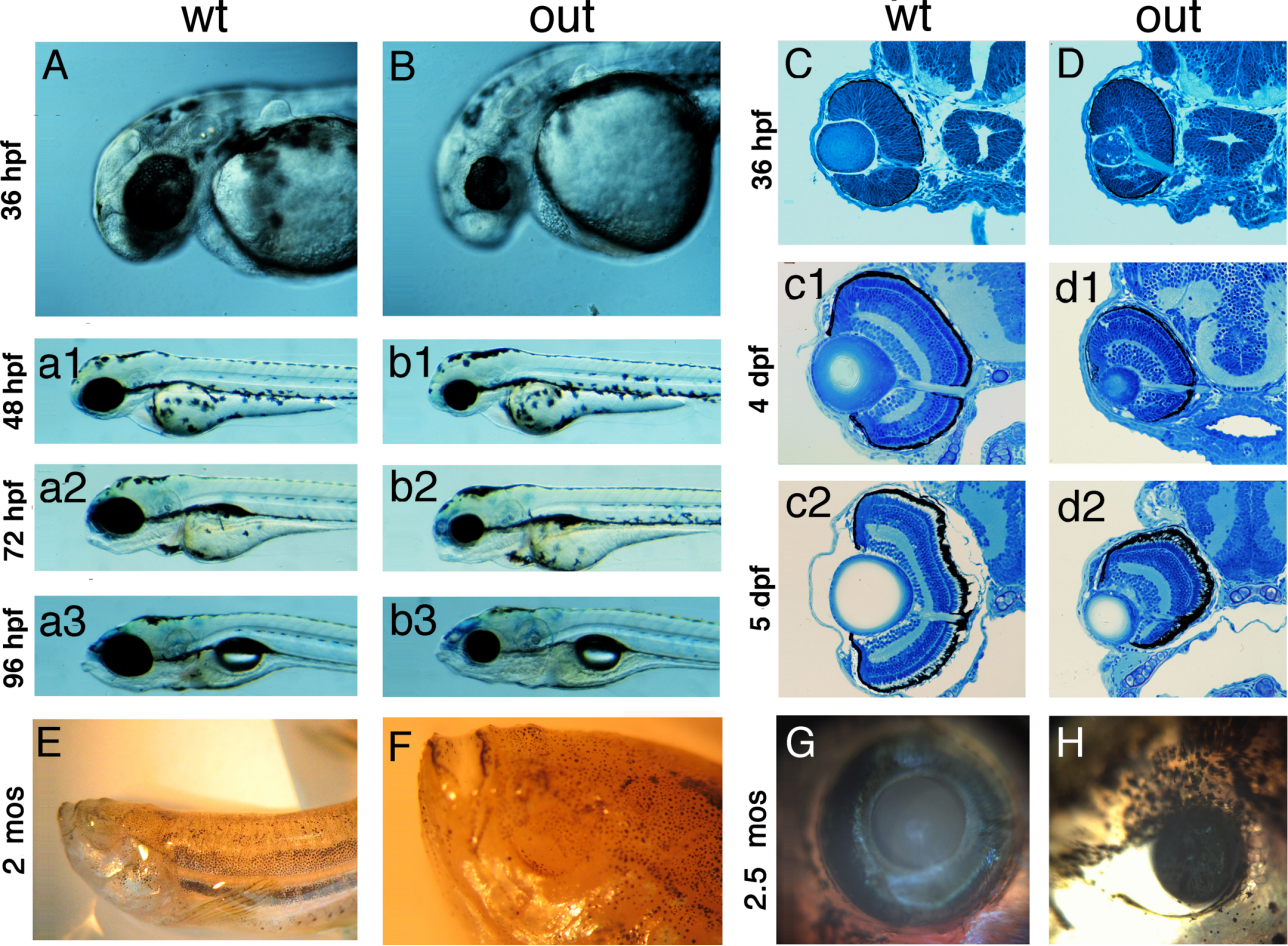
**Phenotype of the zebrafish *out of sight *mutant**. (A - B) Lateral views of wild-type zebrafish (A) and of the *out*^*m233 *^mutant (B) at 36 hpf, 48 hpf (a1 and b1), 72 hpf (a2 and b2) and 96 hpf (a3 and b3). The *out*^*m233 *^mutant is characterized by a severe reduction in eye size. No obvious defects are observed in other organs. (C - D) Transverse plastic sections of wild-type (C) and *out*^*m233 *^mutant (D) retinae at 36 hpf, 4 dpf (c1 and d1) and 5 dpf (c2 and d2). Although the retina and lens are grossly reduced in size in *out*^*m233 *^mutants, their overall architectures appear largely intact. (E - F) An anophthalmic *out*^*m233 *^homozygous mutant at ca. 2 months of age. An enlargement of the head region is shown in (F). (G - H) A wild-type individual (G) and its microphthalmic *out*^*m233 *^sibling (H) at 2.5 months of age. In A, B, and E-H, anterior is to the left.

The *out of sight *(*out*)^*m233 *^mutant was identified in a large-scale ENU mutagenesis screen [25,26]. Out of several small eye mutants that were isolated in this experiment, *out*^*m233 *^displayed the most severe phenotype already distinguishable during day 2 of development [26] (Figure 1). Pigmentation appears to be darker in *out*^*m233*^, compared to its wild-type siblings at 5 dpf, but no obvious defects are observed in other organs, and some mutant fish differentiate the swim bladder. The majority of mutant individuals die between 5 and 10 dpf, and only a small fraction of animals survive to several weeks of age. These individuals have grossly normal body shapes, but feature very small eyes and in the most extreme cases are anophthalmic (Figure 1E, F). Histological analysis indicates that both the retina and the lens are grossly reduced in size in *out*^*m233 *^mutants (Figure 1). Although the retina is reduced in size by ~50% at 5 dpf, in most mutant individuals its overall architecture appears grossly intact.

### Positional cloning of the *out*^*m233 *^mutation

**Figure 2:**
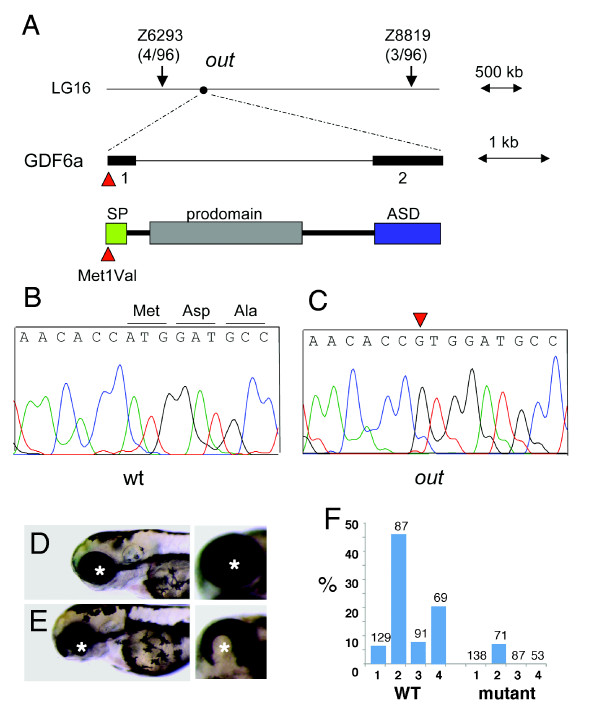
**Positional cloning of the zebrafish *out of sight *mutation**. (A) The *out *locus maps to chromosome 16 between markers Z6293 and Z8819. Sequence analysis of the *gdf6a *gene from this interval identified a mutation affecting its start codon (c.1A > G, p. Met1Val) in mutant (C) but not wild-type (B) individuals. SP = signal peptide, ASD = active signaling domain. (D, E) The overexpression of wild-type (E) but not mutant (D) *gdf6a *causes ventral eye defects. Shown are lateral (left panels) and ventro-lateral (right panels) views of zebrafish larvae at 3 dpf. Anterior is to the left, asterisks indicate the lens. (F) The frequency of ventral eye defects, following the overexpression of wild-type or mutant *gdf6a *in four independent experiments, as indicated on the horizontal axis (1 through 4). Numbers above the bar graph indicate sample sizes.

Mapping analysis revealed that the *out *locus is located on chromosome 16 between markers Z6293 and Z8819 (Figure 2). This region contains around 41 annotated genes, including *gdf6a *(*radar*, *LOC566470*). Sequence analysis of *gdf6a *in the *out*^*m233 *^mutant identified a c.1A > G transition affecting the start codon (p.Met1Val). This is the first ATG in the *gdf6a *transcript, and is preceded by a termination codon 42 bp upstream, confirming that this is the translation start site. The first ATG following the affected start codon is located four base pairs downstream (c.5A), and if used would result in a shift in the open reading frame. However, that ATG might not be used as a translation start site since it is not embedded in a Kozak consensus sequence. Another translation start site that is predicted to be functional by NetStart [27] is located at position c.223A, and its use would produce a protein in the correct reading frame but lacking the first 74 amino acids, including the signal peptide.

To confirm that its defects are indeed responsible for the *out*^*m233 *^mutant phenotype, we overexpressed the wild-type *gdf6a *in the progeny of crosses between *out*^*m233 *^heterozygotes. This was accomplished by injecting into embryos a DNA construct containing *gdf6a *under control of a heat-shock promoter. A mutant *gdf6a *gene that contains the p.Met1Val substitution was used in these experiments as a negative control. The overexpression of the wild-type *gdf6a *produced a significant decrease (p = 0.003, chi square) in the frequency of phenotypically mutant animals from ca. 20% (34/171) to 8% (13/154). At the same time, we observed that ca. 9% (14/155) of animals featured one small and one normal size eye. This phenotype was entirely absent in animals treated with the control mutant construct (0/171). We interpret the one eye phenotype as a partial rescue of *out*^*m233 *^eye size defect.

To further test whether the p.Met1Val substitution affects gene function, we overexpressed wild-type and mutant Gdf6 in zebrafish using a heat-inducible promoter. In agreement with previous studies [24], the overexpression of the wild-type Gdf6 construct frequently results in ventral eye defects. In contrast to that, the overexpression of the mutant gene produces ventral eye abnormalities with a much lower frequency (Figure 2D-F). These data are in agreement with the results of *out*^*m233 *^rescue experiments, and indicate that the p.Met1Val substitution affects gene function. Based on these results, we conclude that mutation in the *gdf6a *gene is responsible for small eye phenotype in the *out*^*m233 *^mutant strain.

### Evaluation of cell fate in *out*^*m233 *^retinae

**Figure 3:**
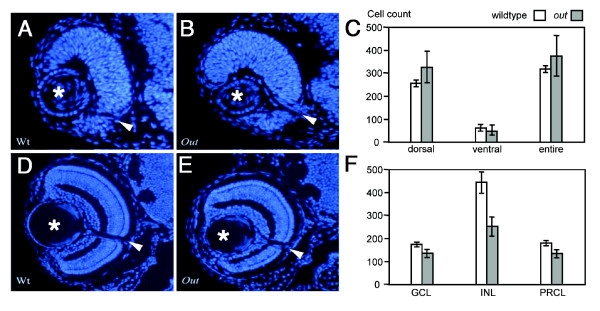
**The number of cells in wild-type and *out*^*m233*^mutant retinae**. (A - B) Hoechst staining of 4-μm plastic sections through the retina of wild-type (A) and *out*^*m233 *^(B) embryos at 36 hpf. (C) Diagram showing the number of nuclei that were counted in the dorsal, ventral, and entire retina. At 36 hpf, cell numbers in mutant embryos are not significantly different from these in wild-type siblings. Cells were counted on sections from 7 and 4 embryos for wild-type and mutant, respectively. (D - E) Hoechst staining of 4-μm plastic sections through the retina of wild-type (D) and *out*^*m233 *^(E) embryos at 96 hpf. (F) Diagram showing the number of nuclei that were counted in the ganglion cell layer (GCL), in the inner nuclear layer (INL) and in the photoreceptor layer (PRCL). By 96 hpf, cell numbers in all these three layers are reduced in mutant embryos, compared to the wild type (for GCL, p < 0.01; for INL and PRCL, p < 0.001; t-test). For both wild-type and mutant, cells were counted on sections from 6 embryos. Asterisks indicate the lens, and arrowheads the optic nerve.

To develop a better understanding of the mutant phenotype, we evaluated the number of retinal cells by counting cell nuclei on sections through wild-type and *gdf6a/out*^*m233 *^retinae at 36 and 96 hpf (Figure 3). At 36 hpf, the number of retinal cells is not statistically different in *out*^*m233 *^mutants, compared to the wild-type. At 96 hpf, cell numbers in all cell layers examined are reduced in mutant embryos, compared to the wild type (p < 0.01 for all cell layers, Student's t-test).

**Figure 4:**
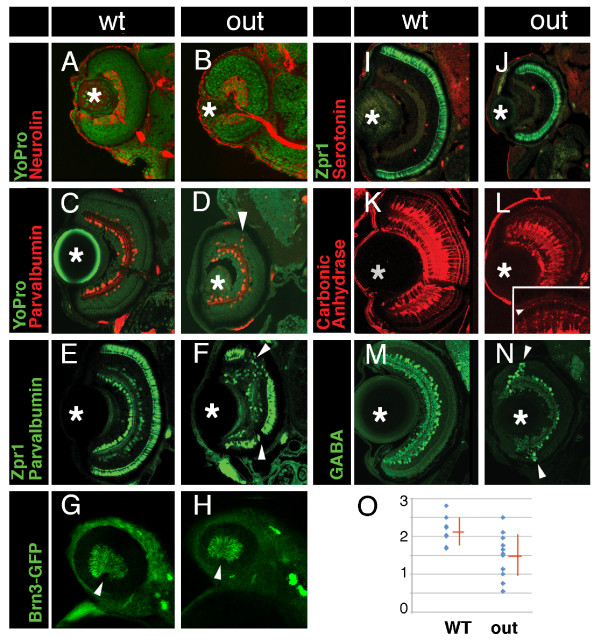
**Cell fate in wild-type and *out*^*m233 *^retinae**. (A-B) Transverse sections through the retina stained with anti-neurolin antibody (red) in wild-type (A) and *out*^*m233 *^(B) embryos. The ganglion cell layer and the optic nerve display grossly normal appearance at 56 hpf. Sections were counterstained with YoPro (green). (C-D). Anti-parvalbumin antibody (red) stains a subset of amacrine cells on transverse sections through the retinae of wild-type (C) and *out*^*m233 *^mutant (D) embryos. A displacement of some amacrine cells towards and occasionally into the photoreceptor cell layer is observed in approximately 50% of *out*^*m233 *^embryos (arrowhead in D). (E - F) Anti-paravalbumin and Zpr1 double immunostaining (both in green) in wild-type (E) and in *out*^*m233 *^mutant (F) embryos. In approximately 50% of the embryos, Zpr1-positive cells are absent in the dorsal and/or ventral regions of the photoreceptor layer (arrowheads in F). (G - H) Images of whole embryos at 42.5 hpf. Ganglion cells are visualized with brn3c-GFP transgene in wild-type (G) and *out*^*m233 *^mutant (H) embryos. Anterior is to the left. (I -J) The Zpr1 antibody stains red and green cones (green signal) while anti-serotonin antibody labels serotonin-positive amacrine cells (red) in wild-type (I) and *out*^*m233 *^mutant (J) embryos. (K-L) Anti-carbonic anhydrase antibody (red) stains Müller glia in wild-type (K) and *out*^*m233 *^mutant (L) embryos. Arrowhead in the inset indicates the apical processes of the Muller glia, which terminate at the outer limiting membrane. (M - N) Anti-GABA antibody (green) stains a subset of amacrine cells in wild-type (M) and *out*^*m233 *^mutant (N) embryos. On the majority of sections, the number of GABA-positive neurons is increased in the retinal periphery of *out*^*m233 *^homozygotes (arrowheads). (O) Graph showing the frequency of GABA-positive cells in the central retina. The number of cells per an arbitrary unit of distance if provided. Fewer GABA-positive cells are observed in the mutant, compared to the wild type (p < 0.01, t-test). Blue dots represent sections. As some sections have the same frequency of GABA-positive cells, the number of dots does not equal the number of sections. n ≥ 4 embryos for both wild-type and mutant. Asterisks mark the lens. (C-F) and (I-N) show retinae at 5 dpf.

To determine whether all major cell classes are present in *out*^*m233 *^retinae, sections through wild-type and mutant larvae were immunolabeled at 2-6 dpf using several antibodies, including anti-carbonic anhydrase for Müller glia [28]; anti-parvalbumin, anti-serotonin, and anti-GABA for subsets of amacrine cells [29]; anti-Pax6 and anti-HuC/HuD for ganglion and amacrine cells [30,31]; anti-Zpr1 for red/green cone photoreceptor cells; anti-rod opsin for rod cells [32,33]; and anti-neurolin for ganglion cells [33] (Figure 4, and data not shown). These experiments demonstrated that all cell classes assayed are present in *out*^*m233 *^embryos in relatively normal proportions, however, some of them display aberrant patterns. For example, in ca. ~50% of *out*^*m233 *^embryos, we observed the absence of Zpr1-positive cells in some regions of the photoreceptor cell layer (Figure 4). This defect frequently correlates with the mislocalization of parvalbumin-positive cells (Figure 4F, compare to the wild type in E). The most interesting defect is present in GABA-positive amacrine cells. On the majority of sections, the number of GABA-positive neurons is increased in the retinal periphery and decreased in the center of the retina (arrowheads in Figure 4N, compare to M, graph in 4O). We have not observed obvious defects in the ganglion cell layer or in Muller glia. The optic nerve displays grossly normal appearance (Figure 4B), and glial cells extend apical processes into the photoreceptor cell layer (Figure 4L, inset).

### Evaluation of cell proliferation and apoptosis during *out*^*m233 *^eye development

**Figure 5:**
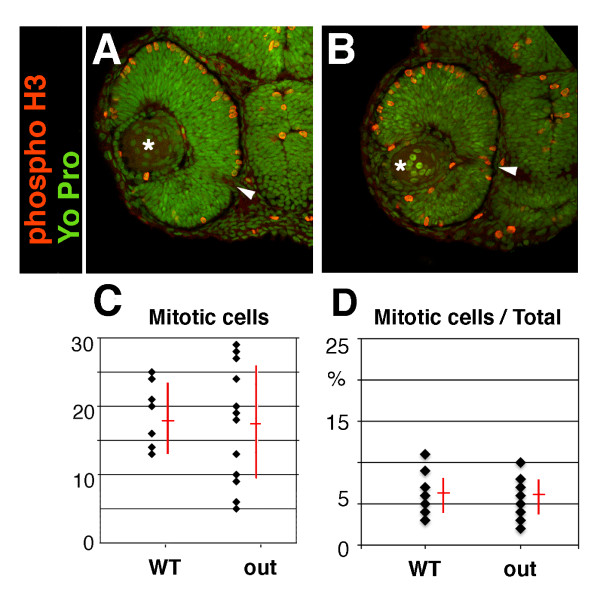
**Cell proliferation in the *out of sight *retina**. (A - B) Immunolabeling of transverse cryosections through the retinae of wild-type (A) and *out*^*m233 *^mutant (B) embryos with anti-phospho-histone H3 (red) antibodies at 36 hpf. Sections were counterstained with YoPro (green). (C) Graph illustrating the numbers of phospho-H3 histone-positive nuclei on sections through wild-type and *out*^*m233 *^mutant retinae as indicated. (D) Graph showing the ratio of phospho-H3-positive cells to all cells. No differences between wild-type and *out*^*m233 *^embryos were identified. In (C - D), black dots represent sections. As some sections have the same number of phospho-H3-positive cells, the number of dots does not equal the number of sections. n ≥ 10 embryos and ≥ 15 sections for both wild-type and mutant samples. Asterisks indicate the lens, and arrowheads the optic nerve. Dorsal is up.

A reduction of organ size can be caused, among other reasons, by changes in the cell cycle, including a premature differentiation of proliferative precursor cells, or increased cell death [34,35]. An increase in the length of G1, S, or G2 phase, for example, would result in a decrease in the frequency of cells positive for phospho-H3, a marker of mitosis. Conversely, an increase in M-phase length would result in an increased occurrence of phospho-H3 positive cells. If, on the other hand, retinal precursor cells are differentiating prematurely, then neurolin, a marker of ganglion cells, the first retinal neurons to become postmitotic [36], is likely to be expressed earlier in the mutant, compared to the wild-type retina. The immunolabeling of mutant and wild-type embryos at 36 hpf with anti-phospho-histone H3 and anti-neurolin antibodies revealed that the levels of phospho-H3 histone-positive (i.e. mitotic) nuclei relative to all nuclei, and the appearance of neurolin-positive ganglion cells in mutant retinae are normal (Figure 5, and data not shown). To further confirm that the timing of neurogenesis is not affected in *out*^*m233 *^mutants, we crossed the *Tg(pou4f3:gap43-GFP) *transgene into the *out*^*m233 *^mutant background. This transgenic line expresses GFP in the ganglion cell layer by 42 hpf [37]. We have not observed consistent differences in the onset of GFP expression between *out*^*m233 *^mutants and their wild-type siblings (n > 20 for both wild-type and mutant; Figure 4H, and data not shown). This indicates that the timing of ganglion cell neurogenesis is unlikely to be defective in *out*^*m233 *^embryos. Based on anti-phospho-H3 staining, it also appears that the length of the M-phase, relative to the rest of the cell cycle is unchanged in the mutant. We cannot, however, exclude the possibility that the overall cell cycle length is affected in *out*^*m233 *^animals.

**Figure 6:**
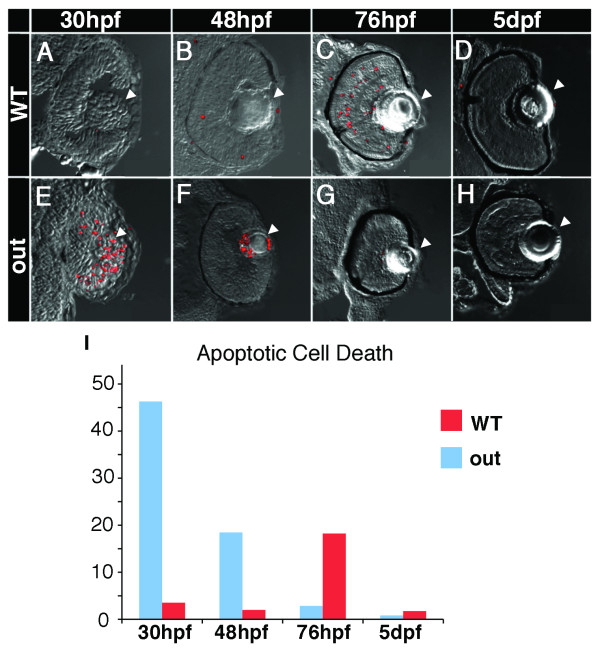
**Apoptosis in *out*^*m233 *^mutant embryos**. (A - H) Transverse cryosections through the zebrafish eye. Apoptosis is detected using the TUNEL assay (red signal). Levels of apoptosis are abnormally high in *out*^*m233 *^mutant (E-H) eyes during early neurogenesis. At 30 hpf, there is an increase of apoptosis in the lens and the retina of mutants (E) compared to the wild-type (A). Apoptosis persists in and around the lens region at 48 hpf (F, compare to B). By 76 hpf, hardly any apoptosis is visible in the mutant eye (G), and a significantly increased amount of apoptosis occurs in the wild-type (C) retina, compared to the mutant. At 5 dpf, hardly any apoptotic cells are found in both the wild type (D) and the mutant (H). (I) The amount of cell death is quantitated. The average number of apoptotic cells per section is provided. Arrowheads point to the lens. n ≥ 9 sections from at least 3 embryos for each time point both for wild-type and mutant samples.

Reduced eye and lens size in mutant embryos may also be due to increased apoptotic removal of retinal cells. To test this possibility, apoptotic cells in wild-type and mutant embryos were visualized by staining with acridine orange (26 hpf, not shown) or TUNEL (30, 48, 72, and 120 hpf). The levels of apoptosis are abnormally high in *out*^*m233 *^mutant eyes during early neurogenesis. Apoptosis in the lens, a normal event in wild-type zebrafish at 24-25 hpf [38,39], persists in mutant embryos. At 30 hpf, an obvious increase of apoptosis is seen in the lens and the retina of mutants, compared to the wild type (Figure 6). Apoptosis persists in and around the lens region in the mutant at 48 hpf. By 76 hpf, on the other hand, there is hardly any apoptotic activity visible in the mutant eye, and a significantly larger amount of apoptosis occurs in the wild-type retina (Figure 6). At 5dpf, few apoptotic cells are detectable both in wild-type and mutant animals. These results suggest that the small eye phenotype is caused by a wave of apoptosis in the mutant eye.

### Mutation analysis of the *GDF6 *gene in patients with microphthalmia

A cohort of 200 unrelated probands with coloboma, microphthalmia and/or anophthalmia was screened for mutations in the *GDF6 *gene. Three heterozygous synonymous changes were identified in 11 patients (c.93C > T/p.Ser31Ser, c.852C > G/p.Ser284Ser, and c.936G > C/p.Ser312Ser). A heterozygous transition (c.955G > A) leading to an amino acid substitution (p.Ala319Thr) was identified in a patient (ID178) with isolated unilateral microphthalmia. This variant was not detected on 362 control chromosomes.

**Figure 7:**
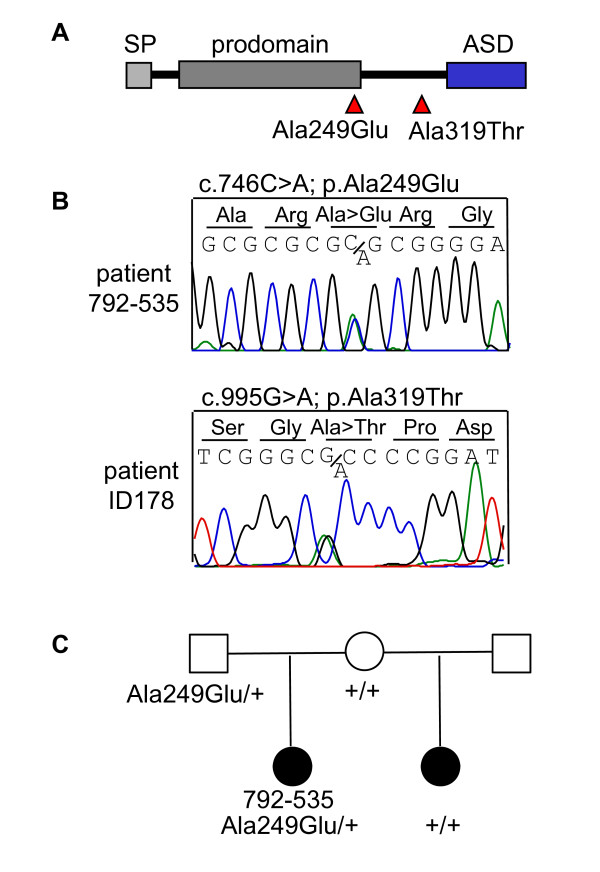
***GDF6 *analysis in patients with microphthalmia, anophthalmia and coloboma**. (A) Two different heterozygous amino acid changes, p.Ala249Glu and p.Ala319Thr, were detected in four unrelated individuals. (B) The sequence of the heterozygous p.Ala249Glu variant in patient 792-535. (C) The affected half-sib of patient 792-535 does not carry the p.Ala249Glu variant, while it was detected in the unaffected father.

In three unrelated patients (ID66, ID136 and 792-535), we identified a heterozygous transition (c.746C > A) leading to an amino acid change (p.Ala249Glu) in the GDF6 prodomain (Figure 7). Besides microphthalmia, patient ID66 has malformed ossicles. Patient ID136 has been diagnosed with cystic fibrosis due to mutations in the *CFTR *gene, microphthalmia, cleft palate, hemivertebrae, hemifacial hypoplasia, a ventriculoperitoneal shunt for hydrocephalus, talipes, growth hormone deficiency and a small pituitary gland. The third patient, 792-535, has a half-sister who was also diagnosed with microphthalmia. The half-sister does not carry the p.Ala249Glu variant, while the carrier father does not have microphthalmia (Figure 7C).

## Discussion

In this study, we characterized the zebrafish *out*^*m233 *^mutant strain and identified a mutation in the *gdf6a *gene affecting the translation start site (p.Met1Val). The size of the retina and the lens are grossly reduced in *gdf6a*/*out*^*m233 *^mutants, but the overall retinal architecture appears largely intact. This is in contrast to other small eye size phenotypes in zebrafish, which often exhibit retinal defects in lamination or retinal degeneration [40,26,41]. Despite eye size reduction, all cell classes that we assayed for are present in *out*^*m233 *^mutants, although some display variable abnormalities. In ~50% of *out*^*m233 *^embryos we observed partial absence of the photoreceptor cell layer in the dorsal and/or ventral regions of the retina. In the regions of photoreceptor absence, we observed a displacement of amacrine cells towards and occasionally into the photoreceptor cell layer. Another interesting defect is present in GABA-positive neurons. These cells are present in abnormally large numbers at the retinal margin at 6 dpf. As neurogenesis continues at the margin of the retina throughout the lifetime of the organism and the quantity of GABA-positive cells decreased in the central retina, this phenotype is most likely transient. As postmitotic GABA-positive neurons near the marginal zone become older, their excess is most probably eliminated by apoptosis.

Our studies suggest that a major contributing factor to the small eye and lens size is deregulated apoptosis. Apoptosis is a normal event during the development of the zebrafish retina, normally occurring around 24 to 25 hpf [38]. The increased apoptosis in the *out*^*m233 *^mutant is evident at 26 hpf and persists until at least 48 hpf, suggesting that retinal precursor cells which normally contribute to the neural retina and lens are being eliminated through apoptosis, which results in a reduction of cell numbers in all retinal layers by 72 hpf.

While this manuscript was in preparation, the small eye size in another zebrafish mutant (*dark half*^*s327*^), was attributed to a nonsense mutation in the *gdf6a *gene [24]. The authors of this work show that *gdf6a *is necessary to induce dorsal fate in the retina. Loss of *gdf6a *prevents specification of the retinal ganglion cells with dorsal identity and prevents innervation of the ventral tectum. *Gdf6a *activates the expression of known dorsal markers (*bmp4*, *tbx5*, *tbx2b*, *efnb2*) and represses the expression of ventral fate determinant, *vax2*. Similar to our observations in the *out*^*m233 *^mutant, the retina of the *dark half*^*s327 *^homozygotes features a transient increase in cell death [24]. Lamination defects have not been reported.

Previous studies have examined the effect of GDF6 loss of function on eye development by morpholino antisense oligonucleotide (MO) knockdown in Xenopus and zebrafish [13,22]. A striking reduction of eye size was observed in Xenopus embryos [22]. In zebrafish, MO injection resulted in highly variable ocular anomalies including ventral colobomata, persistent dorsal-retinal groove, microphthalmia, and even anophthalmia [13]. The retina was disorganized after MO knockdown in Xenopus and zebrafish, and immunolabeling with anti-XAP-1 and anti-islet-1 antibodies in Xenopus did not detect any photoreceptor, ganglion or amacrine cells [13,22]. This is in contrast to our findings in the *out*^*m233 *^mutant, where retinal lamination is largely normal and all cell classes are present.

MO knockdown of *gdf6a *in zebrafish recently identified the presence of skeletal anomalies (curled or kinked tails), although their prevalence was much lower than that of ocular anomalies [14]. Axial abnormalities were also seen in Xenopus tadpoles following morpholino injection [21]. In these studies, MO knockdown of *gdf6a *did not always lead to a mutant phenotype, as significantly reduced levels of correctly spliced *gdf6a *mRNA were identified in phenotypically unaffected morphant embryo pools [14]. In contrast to these findings, other than eye size reduction, no obvious defects are observed in the external appearance of *out*^*m233 *^mutants, including its craniofacial skeleton. The external morphology of *dark half*^*s327 *^mutants was also inconspicuous, with the exception of smaller eyes [24]. Similarly, we also did not observe any defects in circulation and axial vasculature in the *out*^*m233 *^mutant, although these were reported after MO-knockdown of *gdf6a *in zebrafish [13,42,43]. The differences observed between MO-injected embryos and the *out*^*m233 *^and *dark half*^*s327 *^mutant strains might be attributed to a toxic effect of the MOs or to non-specific interference of anti-*gdf6a *morpholinos with other genes. Non-target related phenotypes including neuronal cell death have been reported for 18% of MOs, even when relatively low MO concentrations are used [44]. Mistargeting by MOs could be caused by the simultaneous inactivation of an essential gene with serendipitous homology, or perhaps by some unexpected chemical contaminants found in a small fraction of MO syntheses [44]. Such nonspecific effects of morpholino knockdown experiments can be usually excluded via rescue experiments [45,46]. In the case of *gdf6*, rescue experiments are difficult to perform as the overexpression of this gene results in the ventralization of the entire embryo [22,21]. An alternative explanation for the discrepancy could be that the *out*^*m233 *^mutant may be a hypomorph that retains some residual *gdf6a *function. This, however, seems unlikely since the *out*^*m233 *^mutation disrupts the translation start site, which will either abolish protein synthesis (if the ATG at position c.5A is used) or produce a protein lacking the signal peptide (if the ATG at position c.233A is used). Such a truncated protein is unlikely to be transported to the endoplasmic reticulum and Golgi for posttranslational modifications, which include two proteolytic cleavage steps[47]. Another defect in the zebrafish *gdf6a *gene was previously reported to result in a short body axis, a reduction of head structures, and death by 48 hpf [48]. That mutant, however, carries a large deletion of many genes, which could explain the more severe phenotype.

In a previous study, a segmental deletion encompassing the *GDF6 *gene was identified in a patient with bilateral chorioretinal colobomata [13]. The patient exhibited multiple developmental defects, including neurodevelopmental impairment, bilateral soft-tissue syndactyly of the toes, and an atrial septal defect. Besides *GDF6*, 30 other genes are also lost in the deletion, which could contribute to the range of developmental defects seen in this patient.

Mutations in *GDF6 *have also been recently reported in patients with Klippel-Feil syndrome, a complex skeletal disorder characterized by congenital fusion of vertebrae within the cervical spine [21]. A chromosomal inversion segregated with the disease in a 5-generation family exhibiting an autosomal dominant inheritance pattern. Besides fusion of the vertebral bodies and laminae, the affected family members had scoliosis of the thoracic and lumbar spine, fusion of the carpal and tarsal joints, and restricted elbow movement. No DNA loss was evident at the inversion boundaries, and no coding genes appeared to be disrupted. The proximal inversion breakpoint was located 623 kb 3'of GDF6. The authors suggest that the disease is associated with *GDF6*, perhaps by disruption of long-range enhancer elements, since the skeletal anomalies in this family correspond with the phenotype observed in *Gdf6 *knockout mice [23]. In these mice, defects in joint, ligament, and cartilage formation were seen in the wrist, the ankle, the middle ear, and the coronal suture between bones in the skull [23]. Analysis of *GDF6 *in 127 individuals with Klippel-Feil syndrome identified two missense variants, p.Ala249Glu and p.Leu289Pro [21]. No ocular defects were described in these patients [21]. The two variants were not identified on 708 control chromosomes [21].

In a recent study, 489 patients with ocular anomalies and 81 patients with vertebral segmentation defects were screened for *GDF6 *mutations [14]. The authors identified four amino acid substitutions associated with ocular phenotypes, two with a skeletal phenotype, and one alteration (p.Ala249Glu) was identified in 3 probands who either had ocular or skeletal phenotypes. These variants were not detected on at least 366 control chromosomes. For three variants, segregation analysis showed the presence of the alteration in an unaffected parent indicating incomplete penetrance. The effect of two amino acid substitutions, p.Ala249Glu and p.Lys424Arg, were evaluated with SOX9 reporter gene assays and Western blot analysis. Mutant GDF6 was less effective in activating SOX9 reporter gene activity compared to wild-type GDF6. By Western blot analysis it was shown that the level of secreted mature GDF6 was reduced with p.Ala249Glu and p.Lys424Arg [14]. Screening of 50 patients with ocular anomalies recently identified *GDF6 *mutations in four (8%) patients[15]. Three of these patients carried the p.Ala249Glu variant.

In this study, we identified two different amino acid substitutions, p.Ala249Glu and p.Ala319Thr, in four (2%) of 200 patients with ocular malformations. Although both variants are conserved among several species, neither is conserved in zebrafish *gdf6a*. Therefore it was not possible to perform rescue experiments in the *out*^*m233 *^mutant to determine the pathogenic potential of these variants. Interestingly, the p.Ala249Glu variant has been associated both with Klippel-Feil syndrome and with ocular anomalies [21,14,15]. The variant was detected on 8 of a total of 1478 chromosomes of patients with ocular malformations [13-15], and not on a total of 1074 control chromosomes [14,21] (Fisher's exact test, *p*-value 0.01), strongly suggesting that it is pathogenic. Parents carrying this variant are asymptomatic, suggesting that *GDF6 *sequence alterations are likely to contribute to the phenotype, but are not the sole cause of the disease. This is confirmed by the fact that the affected sib of one of the patients in our study does not carry the p.Ala249Glu variant, indicating that other factors contribute to the disease. Besides microphthalmia, one patient in our study carrying the p.Ala249Glu variant has malformed ossicles. Interestingly, middle ear defects were also observed in *Gdf6*^*-/- *^mice, suggesting that the p.Ala249Glu variant could contribute to the ocular and middle ear defects in this patient. Finally, another microphthalmic patient in our study who carries the p.Ala249Glu variant has several skeletal anomalies, including cleft palate, hemivertebrae and talipes. Microphthalmia in combination with cleft lip and palate have recently been described in another patient carrying the p.Ala249Glu variant [15], suggesting that the extra-ocular defects might also be attributed to the p.Ala249Glu variant.

## Conclusions

This study confirms the essential role of GDF6 in the regulation of vertebrate eye size. The reduced eye size in the zebrafish gdf6a/*out*^*m233 *^mutant is likely to be caused by a transient wave of apoptosis at the onset of neurogenesis. Amino acid substitutions in *GDF6 *were detected in four patients with microphthalmia. In two patients different skeletal defects were observed, suggesting pleitrophic effects of p.Ala249Glu. Variable expressivity and penetrance suggest a complex non-Mendelian inheritance pattern where other genetic factors may influence the outcome of the phenotype.

## Methods

### Animals

The maintenance and breeding of zebrafish strains and staging of embryonic development were performed as described previously [49,50]. All animal protocols were approved by the MEEI and Tufts University animal care committees. Embryos were observed using an Axioscope microscope (Zeiss, Thornwood, NY) or a Leica (Deerfield, IL) MZ12 dissecting microscope. Images were recorded with digital cameras, and processed using Adobe Photoshop software. The *out*^*m233 *^mutant strain was originally recovered in a large-scale N-ethyl-N-nitrosurea (ENU) mutagenesis screen [25,26]. To visualize ganglion cells, we used the *Tg(pou4f3:gap43-GFP) *trangenic line [37].

### Histology

Zebrafish larvae were raised to the desired age and fixed in 4% paraformaldehyde (PFA) in PBST overnight at 4°C. Embedding, sectioning, and staining were performed as described previously [51,52]. Sections were examined using an Axioscope Microscope (Zeiss) and images were recorded using an AxioCam digital camera (Zeiss).

### Positional cloning

A map cross was set up between heterozygous G0 carriers of the *out*^*m233 *^allele (AB genetic background) and wild-type WIK strain homozygotes. To determine the position of the *out *locus in the genome, we used a panel of 96 F2 diploid embryos obtained via incrossing of F1 animals.

Bulk segregant mapping analysis was performed by the Mutant Mapping Facility at the University of Louisville on DNA isolated from the mutant embryos http://www.biochemistry.louisville.edu/zfmapping/index.html. Positional candidate genes were selected based on the zebrafish genome sequence (Ensembl Database, Sanger Center UK, and UCSC Genome Browser, University of California at Santa Cruz). Search for mutations was performed by RT-PCR of RNA isolated from wild-type and *out*^*m233 *^embryos, and by PCR on genomic DNA. PCR products were purified using the QIAquick PCR purification kit (Qiagen) and analyzed by direct sequencing. The effect of the mutation, which affects the translation start site, was evaluated with NetStart 1.0 [27].

### Rescue and overexpression experiments

The wild-type *gdf6a *gene was previously cloned into the pNG6 vector under the control of a heat-shock promoter [24]. The p.Met1Val mutation was introduced by site-directed mutagenesis of the wild-type *gdf6a *construct with phusion DNA polymerase (Finnzymes, Espoo, Finland). The wild-type and mutant constructs were injected into zebrafish embryos at the one-cell stage using standard approaches [53]. Expression was activated via a 1 hour heat shock at the 12 somite stage. The eye phenotype was evaluated at 3 and 5 dpf.

### Staining of nuclei

To count cells in retinal layers, wild-type and mutant embryos were fixed in 4% PFA at 36 and 96 hpf, and embedded in JB-4 resin (Polysciences), according to the manufacturer's instructions. Subsequently, 4-μm sections were collected on microscope slides, immersed in Hoechst 33258 solution (Molecular Probes; 1 mg/ml in PBS) for 10 min, and washed in PBS for 1 h. Alternatively, YoPro (Molecular Probes) was diluted 1:5,000 in PBST and used to stain frozen sections for 10 min. Sections were analyzed using UV illumination.

### Analysis of cell death

Two methods were used to evaluate apoptotic cell death: acridine orange staining and TUNEL. To apply the first method, embryos were dechorionated, and placed in 5 mg/ml acridine orange (acridinium chloride hemi-zinc chloride; Sigma) in E3 medium [54] for 30 min. Embryos were then washed in E3 medium and viewed using UV illumination on an upright microscope. For TUNEL detection of cell death, zebrafish larvae were fixed in 4% PFA, cryoprotected in sucrose, and cryosectioned at 14 μm. Following two washes, 10 min each, in 50 mM PBS (pH 7.4), sections were treated with Proteinase K (Roche Applied Sciences, Indianapolis, IN) for 10 min at the concentration of 2 μg/ml in 50 mM Tris/HCl buffer, pH 8. Subsequently, sections were rinsed 3 times in 50 mM Tris/HCl buffer, incubated with 70% ethanol/30% acetic acid solution at -20°, washed 5 times, 5 min each, in 50 mM Tris/HCl buffer, and treated with blocking solution, containing (v/v in PBST) 10% normal goat serum, and 0.5% Triton X-100 (30 min, room temperature). Finally, sections were incubated with TUNEL reaction mixture (Roche) at 37°for several hours according to manufacturer's instructions. To detect apoptotic nuclei, sections were rinsed in 50 mM PBS for 10 min, coverslipped, and analyzed on a Leica SP2 confocal microscope equipped with a 40x lens. Digital images were processed with Adobe Photoshop Software and used to obtain counts of apoptotic cells.

### Immunohistochemistry

Antibody staining was performed on frozen sections as described in previous publications [51,52,29]. The following primary antibodies and dilutions were used: rabbit anti-phospho-H3 histone (1:200, Upstate Biotechnology); mouse monoclonal anti-neurolin (1:25, Zebrafish International Resource Center); rabbit polyclonal anti-carbonic anhydrase (1:250, gift of Paul Linser, Whitney Laboratory, St. Augustine, FL); mouse monoclonal anti-parvalbumin (1:250, Chemicon); mouse monoclonal anti-HuC/HuD (1:20, Molecular Probes); mouse monoclonal Zpr1 (1:250, Zebrafish International Resource Center); rabbit polyclonal anti-serotonin (1:250, Sigma); rabbit polyclonal anti-tyrosine hydroxylase (1:250, Chemicon); rabbit polyclonal anti-GABA (1:500, Sigma); and rabbit polyclonal anti-PAX6 (1:200, Covance). The anti-serotonin and anti-Pax6 antibodies required citrate treatment prior to blocking in normal goat serum [29]. After staining, sections were imaged using Leica SP2 confocal microscope equipped with a 40x lens. Digital images were processed with Adobe Photoshop Software.

### Patient samples and mutation analysis

Informed consent was obtained from all participants in this study in accordance with the local Ethic Boards in the respective institutions where they were examined. Blood samples were collected, and genomic DNA was isolated by standard protocols. The coding region and splice junctions of the two exons of *GDF6 *were screened in 200 patients with coloboma, microphthalmia or anophthalmia by automated sequencing [21]. PCR products were treated with ExoSAP-IT (USB) or purified with 96-wells NucleoFast purification plates (Machery-Nagel) before automated sequencing. All mutations and polymorphisms were confirmed by a second round of PCR amplification.

## Authors' contributions

AIdH carried out the molecular genetic studies and drafted the manuscript. JB and PK carried out the histological and immunohistochemical studies. TB, EIT, NKR, and AS provided patient samples. JM conceived of the study, participated in its design and coordination and helped to draft the manuscript. All authors read and approved the final manuscript.
